# Walking with UAN.GO Exoskeleton: Training and Compliance in a Multiple Sclerosis Patient

**DOI:** 10.3390/neurolint13030042

**Published:** 2021-08-23

**Authors:** Gianluca Sesenna, Cecilia Calzolari, Maria Paola Gruppi, Gianluca Ciardi

**Affiliations:** 1 U&O, 29017 Fiorenzuola d’Arda, Italy; gianluca.sesenna@uando.it; 2 Degree Course of Physiotherapy Student, Parma University, 43121 Parma, Italy; cecilia.calzolari@studenti.unipr.it; 3 Azienda USL, 29121 Piacenza, Italy; mariapaola.gruppi@unipr.it; 4 Degree Course of Physiotherapy, Parma University, 43121 Parma, Italy

**Keywords:** multiple sclerosis, exoskeleton, rehabilitation, walking

## Abstract

Background: Multiple sclerosis is a progressive neurodegenerative disease that affects myelin in the central nervous system. It is complex and unpredictable and occurs predominantly in young adults, causing increasing disability and a significantly lower quality of life. Recent studies investigated how rehabilitation training through the use of a robotic exoskeleton can influence walking recovery in patients with a serious neurological disease. Aim: The purpose of this study was to analyze the first approach of a multiple sclerosis patient to a robotic exoskeleton for the lower limbs, in order to assess the effectiveness of the protocol on walking ability, adaptability of the device, level of appreciation, variations in parameters related to walking, and fatigue perception. Methods: This study was conducted on a 71-year-old male diagnosed with primary progressive multiple sclerosis since 2012, with an EDSS score of 6. The patient underwent a cycle of 10 sessions of treatment with the exoskeleton for the lower limbs, the UAN.GO, lasting 1 h 30 min. Pre- and post-treatment evaluations were carried out with the 6 min walking test, the Fatigue Severity Scale, the Short Form-36 Health Survey, and a Likert scale for review. During each session, blood pressure, heart rate, and peripheral saturation were monitored; in addition, the perception of fatigue by the Borg scale was studied. Result: A comparison between the initial and final evaluations showed improvements in the walked distance at 6 MWT (T_0_ = 53 m/T_1_ = 61 m). There was a positive trend in saturation and heart rate values collected during each session. Further improvements were found by the Borg scale (T_0_ = 15/T_1_ = 11). Discussion: The data collected in this case report show promising results regarding the treatment of multiple sclerosis patients with the UAN.GO exoskeleton, with benefits on both motor performance and vital parameters.

## 1. Background

Multiple sclerosis (MS) is a chronic inflammatory disease that affects the central nervous system (CNS) [1] associated with an inflammatory autoimmune component and a neurodegenerative component [2]. MS attacks and damages myelin, causing a delayed transmission of nerve impulses. Motor, sensory, visual, and autonomous systems are involved; MS pathogenesis is still unclear. Recent articles underlined some risk factors for MS onset [3]: in particular, smoking, childhood obesity, and low vitamin D levels are now under authors’ attention. Another interesting research field for MS onset is that about immunodeficiency and viral disease: some authors sustain the hypothesis that Epstein–Barr virus, influenza, and mononucleosis virus could be involved in MS pathogenesis [4,5,6]. To date, 2.3 million people worldwide have been diagnosed with multiple sclerosis [7]. Symptoms linked to MS are very varied, due to the dissemination of lesions, their severity, and the degree of repair as a result of myelin damage and may include: psychic disorders, vision disorders, motor disorders, and symptoms attributable to a lesion of the brain stem such as dizziness, diplopia, or balance deficit [2]. The movement system represents one of the main targets of MS, with the possible appearance of spasticity, walking disorders, coordination deficit, postural instability, muscle weakness, easy patient fatigue, and a high degree of dependence in the execution of ADL [8].

Physiotherapy treatment of MS represents an extremely complex moment for these patients; recent evidence recommends focusing on a multidisciplinary approach that includes physiotherapy, occupational therapy, orthotic adoption, physical therapy, hippotherapy, vibration-based therapy, acupuncture, speech therapy, targeted therapy for upper limb disorders, and muscle tone control [5]. In detail, a Cochrane group RCT review has been published about physiotherapy intervention for MS patients: strong evidence for exercise therapy in terms of muscle function, exercise tolerance, and mobility-related activity was demonstrated; despite this, no specific profile of a high-evidence protocol emerged [9]. A wide range of physiotherapy techniques have been tested in MS patients with contrasting results: balance-based exercises have a low significant effect [10], such as yoga therapy [11] and flexibility exercises [12], while high-intensity interval training seems to be more effective on the fitness ability in mild MS patients [13].

A field of interest for physiotherapists is gait rehabilitation of MS: the recent literature has strongly underlined deficit and pathological patterns for MS patients, in order to describe a correct approach. In particular, a recent systematic review and meta-analysis by Comber [14] described the typical deficits in MS patients’ gait (EDSS scoring from 1.8 to 4.5) compared to the general population:

− Significantly lower gait speed [15,16,17,18,19,20];
− Reduced cadence of gait [15,17,19,20];
− Increased stride and step length [16,17,21];
− Increased double support time [15,17,19,20];
− Reduced swing phase [15,19,20].

In this landscape of increasing scientific interest, one of the new rehabilitation boundaries being explored is gait robotic rehabilitation through specific aids. The enormous potential of robotic devices has been partially exploited in many industries, where robots carry out various repetitive tasks; starting from this experience, there has been a need to broaden their field of use in order to integrate them into daily life. A promising direction is represented by the exoskeletons that have been developed in recent decades, which enclose the human limbs to increase their existing capabilities or replace compromised ones [22,23,24]. This innovative robotic rehabilitation program is based on the hypothesis that repetitions of oriented movements can stimulate reorganization at the spinal cord level. In particular, this type of treatment allows increasing the total duration of training, reducing the need for assistance by therapists. Nevertheless, a specific training protocol has not yet been recommended, nor has a range of disabilities within which this type of device can be used; currently available studies are mainly focused on patients with spinal injuries (to be included in myelin injury studies). In this report, a patient with MS was monitored while performing robotic path training using the UAN.GO exoskeleton.

## 2. Materials and Methods

A single patient case report was conducted; inclusion criteria were: adult with MS diagnosis, and a strong motivation to achieve walking improvements. Exclusion criteria: psychiatric disorders, and spasticity (scoring more than 3 on the Ashworth scale).

A male patient, 71 years old, with a diagnosis of PP MS since 2012 and an EDSS [25] score of 6, was recruited and enrolled in the study, after signing informed consent. He underwent a total of 10 physiotherapy sessions, including an introductory session, of 1.5 h each, performed 2 times a week, in addition to the usual physiotherapy treatment carried out once a week. Having no reference literature on patients with MS, a protocol of an approach to walking previously tested on paraplegic patients was selected to determine whether the exoskeleton and treatment protocol could affect the rehabilitation path in this type of patient. No ethics committee approval was required, as suggested for clinical case reports by the Aven guidelines [26]; the patient signed informed consent before the treatment.

Study timeline: an initial evaluation (T_0_) was carried out, consisting of functional rehabilitation analysis and measurement of anthropometric parameters necessary for the setting of the machine. After the tenth session (T_1_), 6MWT [27], SF36 [28], and FSS [29] were evaluated.

At the end of the treatment, data collected were compared to describe their trends. In addition, vital parameters (SpO2, PA, FC) and perception of fatigue using the Borg scale at the beginning and at the end of each session were monitored [30]. The patient, at the end of the session cycle, was then given a questionnaire, built according to the Likert model, about his liking of the device and the training [31].

The device chosen for the study was the UAN.GO lower limb exoskeleton designed by U&O srl, an Italian startup specialized in the design and production of robotic exoskeletons for medical use, and in the production of aids for the rehabilitation of people with lower limb disabilities and movement disorders. The UAN.GO exoskeleton is a powered lower limb exoskeleton for overground gait training designed to be used in a rehabilitation setting to progress neurorehab patients, certified as a medical device, CE IIA (CEE 93/42), for clinical and personal use. This medical device is a powered lower limb exoskeleton, which provides people with mobility impairments with the opportunity to walk independently. Power is provided by sophisticated motors in the knee and hip joints, and combined with advanced sensors and control strategies, the device allows gait-impaired individuals to stand up and walk again. UAN.GO is equipped with four motorized joints (hips and knees) and four passive joints (ankles and feet). It allows robotized walking training (optimized kinematics due to 8 joints) with weight completely discharged to the ground and guided by a software that facilitates and helps operators in rehabilitating the functions necessary to retrain the gait. It allows “passive” and “active-assisted” training modes thanks to a “full or partial powered support” selectable by a control unit for the joints’ motor power; it can be used in “Assisted Mode” (caregiver selects the movement maps from the on-board touchscreen and activates them via the start/stop push buttons) or in “Autonomous Mode” (caregiver selects the movement trigger positions and the patient autonomously controls the exoskeleton thanks to the movements of their trunk). It allows the execution of step-by-step training to help patients train in the skills necessary to walk, and UAN.GO also allows the execution of stairs. Thanks to the possibility of configuring and customizing the movement maps, the opportunities of activating them in different ways according to the patient’s skill level, and the disruptive device setup times (mechanical-anatomical setup times and device software lower than 2 min), UAN.GO is a robotic rehabilitation device designed to help clinical staff quickly, easily, and effectively during gait rehabilitation.

The device is intended for individuals with complete SCI at levels T4 to L5 (with upper extremity motor function of at least 4/5 in both arms), and incomplete SCI at levels of C7 to T3 (with upper extremity motor function of at least 4/5 in both arms). UAN.GO has also been designed for patients with hemiplegia due to stroke and multiple sclerosis patients, but more generally for all those with lower limb disorders related to neurological pathologies and neurodegenerative diseases with severe gait impairment.

As shown in Table 1, for UAN.GO training, the patient has to meet some inclusion criteria: good mineral density, weight under 100 kg, good general health, ability to use a walker, height between 160 and 195 cm. As well as other robotic orthoses, general contraindications are the presence of fractures, severe spasticity, reduced PROMs (including POA or other nature calcifications), systemic disease, and psychiatric disorders. Table 2 shows UAN.GO training program.

**Figure 1:**
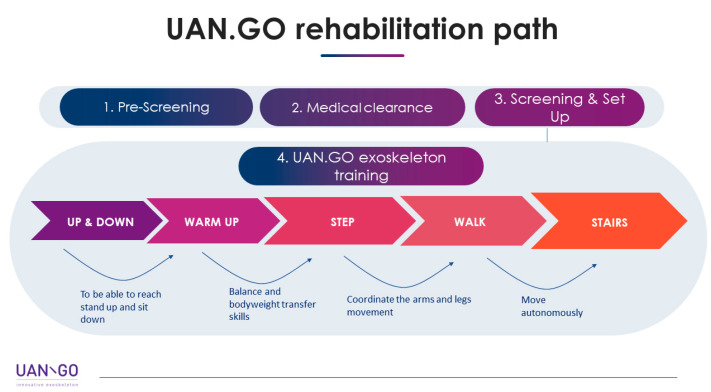
UAN.GO treatment protocol; developed by U&O.

Once the initial screening is completed, the treatment protocol starts with a progressive task-oriented training (Figure 1):

− The first ability to develop while wearing the UAN.GO is standing up and sitting down.
− With the warm-up, the patient learns to load his weight through the exoskeleton, and the skill to transfer it to the other leg.
− The third moment of UAN.GO training is step: joining the acquired abilities, the patient has to stand, transfer the weight through the trunk, and move a leg to 90 degrees flexion.
− Then, the patient is sure to walk (fourth moment of training), reaching a good coordination between the legs and arms.
− The last point of the rehabilitation protocol is about autonomous movement.

The training protocol was developed in accordance with the previous literature [32,33].

## 3. Results

At the end of the sessions, final evaluations were carried out using the same means and methods as the initial ones. A significant improvement was found in motor performance during the execution of the 6MWT, and also in the perception of fatigue during individual sessions, with a decrease in Borg scores. A positive trend regarding pressure, saturation, and heart rate values emerged from data analysis; furthermore, a slight improvement was noted in some subscales of the SF-36 and FSS. The following are the tables about the results of the initial and final evaluations and their reference graphs.

**Figure 2:**
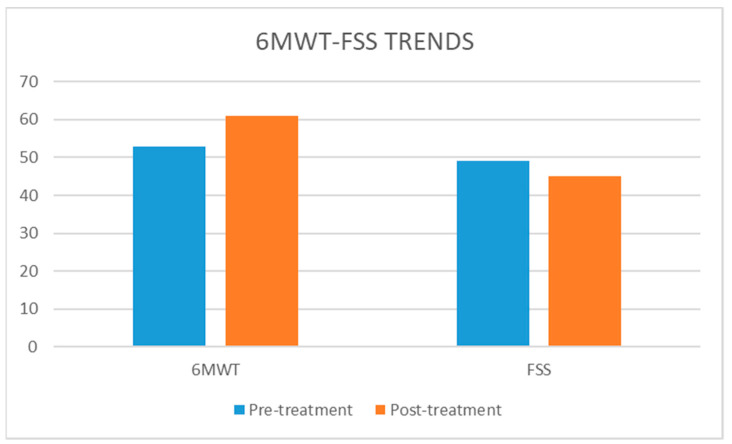
Trend of 6mwt and fss scoring.

In detail, Figure 2 shows an increased 6MWT distance, from the first evaluation (53 m) to the last one (61 m), and the FSS scoring, with a pre/post decrease of 4 points—Figure 2.

**Figure 3:**
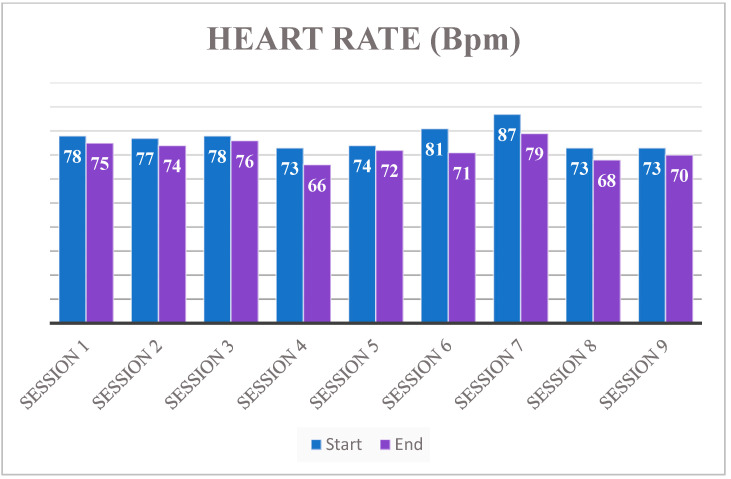
Heart rate trend.

**Figure 4:**
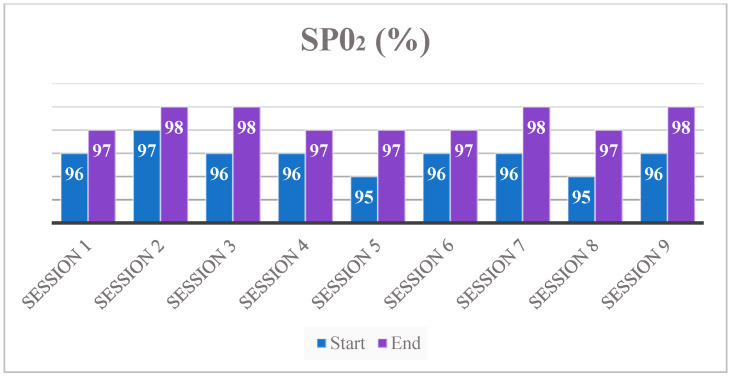
Trend of peripheral saturation (Sp0_2_).

Table 3, instead, shows the blood pressure trend, with an increase of 5/10 MmHg at the end of each training session; these data, with the hearth rate trend (Figure 3) and stable peripheral SpO_2_ (mean value at the end of each training session 98%—Figure 4), show an overall training ability by the UAN.GO.

**Figure 5:**
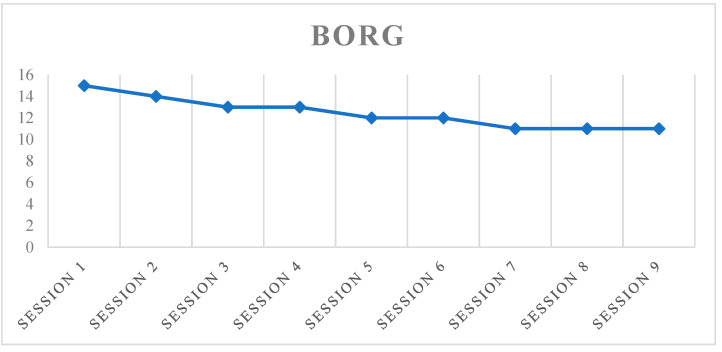
Trend of the Borg score.

Further confirmation of this hypothesis was a good trend in the Borg scoring from the first observation (15 points) to the last one (10 points), as shown in Figure 5.

**Figure 6:**
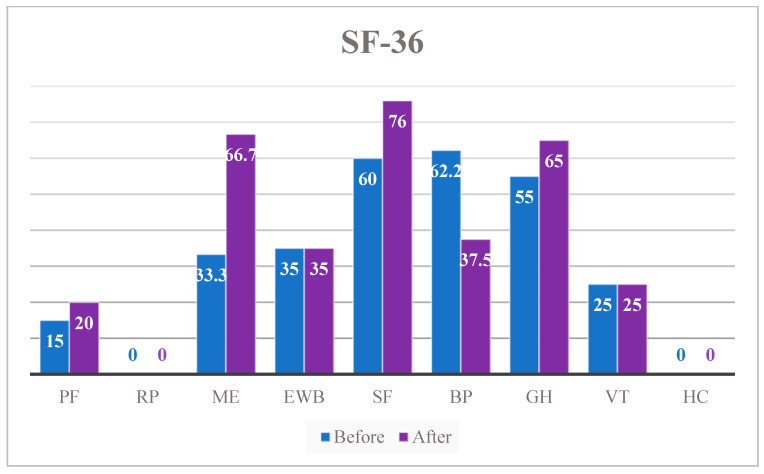
Trend of the SF-36.

Table 4 and Figure 6 finally, describe the SF-36 trend from the first to the last training session; most improvements were about emotional role scoring (+33 points), pain detected (−24 points), social functioning (+16 points), and general health (+10 points). Minimal or no change was reported on physical function, role physical, emotional well-being, energy/fatigue, and health change domains.

## 4. Discussions

The present article was compliant with the CARE guidelines [34]. Walking problems have a strong negative impact on the quality of life of patients with multiple sclerosis, not only in terms of motor appearance but also in relation to participation in social activities [35]. In this study, the comparison between the initial and final assessments showed a promising result regarding the treatment of patients with primary progressive multiple sclerosis with the UAN.GO exoskeleton, with benefits in both motor performance and vital parameters. The device and treatment protocol were well tolerated by the patient, who reported his feedback to a physiotherapist, to better adapt the exoskeleton and treatment to his needs. The liking questionnaire administered at the end of the session cycle highlighted how the UAN.GO exoskeleton fits comfortably and does not generate painful pressure points. In general, the patient obtained some benefits from the treatment protocol with the UAN.GO. First of all, the trend of saturation and heart rate values showed a progressive adaptation to the proposed exercise, confirming the reconditioning capacity that exoskeletons can produce in patients with walking difficulties. The data are further highlighted by the 6MWT profile, and perceived fatigue on the Borg scale. The patient also showed a clear increase in his well-being and quality of life: the SF-36 scoring showed that at the end of the sessions, the items most improved were those relating to the pain experienced. All these findings seem in line with the precedent literature, which shows similar benefits in treatment with an exoskeleton for walking [36,37,38].

However, some limitations emerged: The first approach to this type of robotic device is not immediate, and a treatment protocol developed for more sessions is needed. The patient therefore reported a need to extend the treatment period, in order to increase his safety in using the device and improve its performance. Despite this, on analyzing the data obtained from the comparison of the Borg values, a progressive decrease in the perception of fatigue was noted. This decrease was also due to the reduction in fear related to improved safety in the use of the device; these data are compliant with recent works in the literature [39,40,41,42,43]. Regarding motor performance, on studying the data obtained from the comparison between the first session and the end of treatment, an improvement was observed in 6MWT, in accordance with the present literature [44,45,46]. There was also a positive trend in saturation and heart rate values, detected within each session, attributable to the physical activity performed. There were no significant improvements in perceived fatigue assessed by the FSS scale. The literature on the evaluation of fatigue symptoms in this type of treatment is poor. Several studies showed significant improvements [47,48], while others do not report any type of variation [49,50]. This heterogeneity in the results can be attributed to several aspects including: variety among treatment protocols and devices on the market, different levels of disability, and the poor existing literature, which does not allow identifying of a precise target of patients.

An in-depth analysis of perceived quality of life, measured through the administration of the SF-36 questionnaire, showed that most significant improvements were observed in emotional problem limitations (RE) and pain reduction (BP); slight differences were obtained in social function (SF) and general health (GH). These improvements in quality of life, although slight, reveal that specific treatment could have a positive impact on patients’ subjective quality of life. This highlights the need to shape rehabilitation interventions around the individual needs of each subject. The results obtained in this short treatment protocol, even if statistically irrelevant, show how training with an exoskeleton positively influenced different areas of the daily life of a patient with multiple sclerosis. Our hypothesis is that extension of the treatment period would have allowed increasing the results. Thanks to the promising results obtained thus far, it would also be appropriate to extend the study to a larger number of subjects for a longer period, programming systematic remote follow-up, with the aim of verifying the maintenance of the results over time and ensuring continuous monitoring.

## 5. Conclusions

The rehabilitation path for patients with multiple sclerosis is a crucial moment in the treatment process, as it represents a necessary measure to counteract disease progression and to improve the perceived quality of life. Training with the UAN.GO seemed to return encouraging results in the rehabilitation of a subject with multiple sclerosis, both from a strictly motor point of view and with respect to the reconditioning capacity and quality of life. Our study represents a pilot experience on this type of intervention, and although it has no statistical value, it can certainly open the way to larger trials which demonstrate the real statistical significance of this intervention.

## Figures and Tables

**Table 1:** Inclusion criteria for training with UAN.GO.

Inclusion Criteria	Exclusion Criteria
Upper limbs able to handle a walker	History of severe neurological diseases associated with severe systemic diseases (e.g., infections, circulatory or heart problems, lung problems)
Absence of unconsolidated fractures	Presence of pressure sores
Good general health	Severe spasticity
Height between 160 and 195 cm	Heterotopic ossifications that reduce ROM
Weight not exceeding 100 kg	Spinal instability or pelvic or AAII fractures not healed
Good bone mineral density	Important retractions
	Psychiatric or cognitive problems that can interfere with the correct use of the device

**Table 2:** Training protocol. Up and down: movement of lifting, and then subsequently of sitting, from a special stool; warm-up: march in place; Step: I walk managing one leg at a time; walk: unlike the step phase, movement is not interrupted between one leg and the other.

Session Number	Treatment
0	Explanation of the study Preliminary assessment Setting of the exoskeleton Dressing Up and Down
1	IMPROVEMENT in PRESSURE POINTS DRESSING UP and DOWN
2	Dressing Up and Down Warm-Up
3	Up and Down Warm-Up Pointing exercises to increase device control
4	Up and Down Warm-Up Step
5	Up and Down Warm-Up Step
6	Up and Down Warm-Up Step Walk
7	Up and Down Warm-Up Step Walk
8	Up and Down Warm-Up Step Walk
9	Up and Down Warm-Up Step Walk

**Table 3:** Trend of blood pressure values.

	Start Session	End Session
Session 1	130/70 mmHg	135/80 mmHg
Session 2	135/70 mmHg	135/70 mmHg
Session 3	125/80 mmHg	120/80 mmHg
Session 4	130/70 mmHg	120/70 mmHg
Session 5	135/70 mmHg	140/80 mmHg
Session 6	130/70 mmHg	130/70 mmHg
Session 7	135/60 mmHg	135/70 mmHg
Session 8	110/70 mmHg	120/70 mmHg
Session 9	135/80 mmHg	140/80 mmHg

**Table 4:** Trend of the SF-36.

M.L.	Before	After	Differences
Physical function (PF)	15	20	5
Role phyisical (RP)	0	0	-
Role emotional (RE)	33.3	66.8	33.6
Emotional well-being (EWB)	35	35	-
Social functioning (SF)	60	76	16
Pain (BP)	62.2	37.6	−24.9
General health (GH)	55	65	10
Energy/fatigue (VT)	25	25	-
Health change (HC)	0	0	-

## Data Availability

Not applicable.
